# IL-17A is implicated in lipopolysaccharide-induced neuroinflammation and cognitive impairment in aged rats via microglial activation

**DOI:** 10.1186/s12974-015-0394-5

**Published:** 2015-09-15

**Authors:** Jie Sun, Susu Zhang, Xiang Zhang, Xiaobao Zhang, Hongquan Dong, Yanning Qian

**Affiliations:** Department of Anesthesiology, The First Affiliated Hospital of Nanjing Medical University, 300 Guangzhou Road, Nanjing, Jiangsu 210029 People’s Republic of China; Department of Anesthesiology, The First People’s Hospital of Lianyungang City, Lianyungang, Jiangsu People’s Republic of China

**Keywords:** IL-17A, Lipopolysaccharide, Neuroinflammation, Microglia, Cognitive impairment

## Abstract

**Background:**

Neuroinflammation is considered a risk factor for impairments in neuronal function and cognition that arise with trauma, infection, and/or disease. IL-17A has been determined to be involved in neurodegenerative diseases such as multiple sclerosis. Recently, IL-17A has been shown to be upregulated in lipopolysaccharide(LPS)-induced systemic inflammation. This study aims to explore the role of IL-17A in LPS-induced neuroinflammation and cognitive impairment.

**Methods:**

Male Sprague–Dawley (SD) rats were injected intraperitoneally with LPS (500 μg/kg), and IL-17A expression in serum and in the hippocampus was examined 6, 12, 24, and 48 h later. Then, we investigated whether IL-17A-neutralizing antibodies (IL-17A Abs, 1 mg/kg) prevented neuroinflammation and memory dysfunction in aged rats that received LPS (500 μg/kg) injection. In addition, the effect of IL-17A on microglial activation in vitro was determined using ELISA and immunofluorescence.

**Results:**

LPS injection increased the expression of IL-17A in serum and in the hippocampus. IL-17A Abs improved LPS-induced memory impairment. In addition, IL-17A Abs prevented the LPS-induced expression of TNF-α, IL-6 and inflammatory proteins, and of inducible nitric oxide synthase (iNOS) and cyclooxygenase-2 (COX-2) as well as the activation of microglia in the brain. IL-17A Abs also inhibited the expression of amyloid precursor protein (APP) and BACE1 and increased the expression of the synaptic marker PSD95 in the aged rats treated with LPS. In an in vitro study, we found that recombinant IL-17A could simulate microglial activation and increase production of pro-inflammatory cytokines.

**Conclusion:**

Taken together, our results suggest that IL-17A was involved in LPS-induced neuroinflammation and cognitive impairment in aged rats via microglial activation. Anti-IL-17A may represent a new therapeutic strategy for the treatment of endotoxemia-induced neuroinflammation and cognitive dysfunction.

**Electronic supplementary material:**

The online version of this article (doi:10.1186/s12974-015-0394-5) contains supplementary material, which is available to authorized users.

## Introduction

Neuroinflammation plays a key role in neurodegenerative diseases such as Alzheimer’s disease and multiple sclerosis (MS) and in memory impairment [1–3]. The elderly are vulnerable to the adverse effects of injections on cognitive function, and the aging process itself is associated with enhanced neuroinflammatory processes involving polarized microglial responses and the production of pro-inflammatory cytokines, with a bias towards M1 and away from M2 activation states [4, 5]. LPS, an endotoxin isolated from bacteria, stimulates pro-inflammatory cascades by acting through plasma membrane proteins, such as toll-like receptor 4 (TLR4), causing pro-inflammatory cytokines to be produced. Systemic injection of LPS induces neuroinflammation and amyloidogenesis in the hippocampus [6]. LPS-induced neuroinflammation in animal models has also been demonstrated to cause memory impairment [7].

Microglia, the resident immune cells in the brain, execute principal inflammatory feedback in various neurodegenerative conditions of the brain. It has been reported that microglia likely play an important role in either the development of protective immune responses or in the progression of damaging inflammation during central nervous system (CNS) disease states [8]. However, uncontrolled activation of microglia leads to the excessive release of various cytokines, such as tumor necrosis factor-alpha (TNF-α), prostaglandin E2 (PGE2), interleukin-6 (IL-6), nitric oxide (NO), and reactive oxygen species (ROS), which have been implicated in various neurodegenerative diseases. Thus, inhibition of the exaggerated inflammatory response by activated microglial cells helps to attenuate the severity of neurodegenerative diseases [9, 10].

IL-17A is the main member of the IL-17 family of cytokines, which includes five other members, designated IL-17A-F, and is secreted by a subset of T (Th17) cells. Although IL-17A alone is a weak inducer of target genes, it has been shown to synergize with IL-1β, IL-22, IFN-γ, TNF-α, and other cytokines in vivo [11]. Notably, IL-17A is strongly involved in mediating pro-inflammatory responses via the induction of many other cytokines, including IL-6, TGF-β, and TNF-α as well as the induction of chemokines, including IL-8 and monocyte chemotactic protein-1 (MCP-1), in many cell types [12]. IL-17A plays an important role in the active states of autoimmune diseases such as MS, during which patients’ clinical symptoms are exacerbated [13]. Recently, IL-17A has been shown to be upregulated in lipopolysaccharide (LPS)-induced systemic inflammation. The present study aims to explore the role of IL-17A in LPS-induced neuroinflammation and cognitive impairment.

## Materials and methods

### Reagents

Dulbecco’s modified Eagle’s medium (DMEM), 0.25 % Trypsin-EDTA solution, fetal calf serum (FCS), penicillin/streptomycin, and poly-D-lysine were purchased from Gibco–BRL (Grand Island, NY, USA). LPS (Coli 0111:B4) was purchased from Sigma–Aldrich (St. Louis, MO, USA). RIPA buffer and the BCA kit were purchased from Beyotime (Shanghai, China). Rat recombinant IL-17A protein, fluoroshield mounting medium with 4,6-diamidino-2-phenylindole (DAPI), and mouse anti-OX42 monoclonal antibody were purchased from Abcam (Hong Kong, China). The rat IL-17A ELISA kit was obtained from Biolegend (San Diego, CA, USA, Cat. no. 437907). Rat IL-6 ELISA kit (R600B) and TNF-α ELISA kit (RTA00) were obtained from R&D Systems, Inc. (Minneapolis, MN, USA). Rabbit anti-Iba1 and anti-PSD95 polyclonal antibodies were purchased from Abcam (Hongkong, China). Rabbit monoclonal antibodies against BACE1, iNOS, COX-2, GAPDH (14C10), and rabbit polyclonal anti-APP antibody and anti-rabbit secondary antibody were all purchased from Cell Signaling (Boston, MA, USA). A FITC-conjugated goat anti-rabbit IgG antibody was purchased from Santa Cruz (Santa Cruz Biotechnology, USA).

### Animals

Male SD rats aged 18 months were purchased from Jinling Hospital of Nanjing University and used in this study (*n* = 70). All rats were housed in groups of five per cage during the experimental period, with water and food available ad libitum. Ambient temperature of the housing and testing rooms was 22 ± 1 °C. Rats were housed under a 12-h light–dark cycle. The study was approved by the Nanjing Medical University Animal Care and Use Committee, and the experiments were performed according to the Guide for the Care and Use of Laboratory Animals of the National Institutes of Health of the United States.

### Drug administration

#### LPS

To induce a systemic inflammatory reaction for the experimental procedures, LPS from *Escherichia coli* (Sigma Chemical, St Louis, MO, USA; 0111:B4) was diluted in saline and injected intraperitoneally (IP) at a dose of 500 μg/kg. This dose was used for the induction of moderate inflammation [14]. Additionally, it has been reported that this dose is within the range that does not affect motor activity [15]. Control rats were IP injected with saline only.

#### IL-17A antibodies

A mouse anti-rat IL-17A antibody (Sangon Biotech Co., Ltd., China; 1 mg/kg) was diluted in saline, which were specific to IL-17 (Additional file 1: Figure S1), and administered intracerebroventricularly (ICV). A total volume of 3 μl (200 μg/μl) was injected before LPS administration. Thirty minutes before LPS/saline administration, rats were anesthetized with isoflurane (1 %), mounted in a stereotaxic frame, and kept at 37 °C using a heating pad. A burrhole was made to inject into the lateral ventricle at the following coordinates (relative to Bregma): 1.5 mm to the right and 0.8 mm posterior. A 33-gauge needle connected to a 10-μl syringe was then lowered 3.7 mm, and either IL-17A Abs or saline (3 μl) was injected at a rate of 1 μl/min. The needle was then left in place for 2 min before being removed to suture the skin. The rats were then placed on a heating pad to recover. Once the rats had regained normal mobility, they were returned to their home cage with unlimited access to food and water and checked regularly for 12 h to ensure there were no adverse effects from surgery.

### Design and treatment groups

**Fig. 1:**
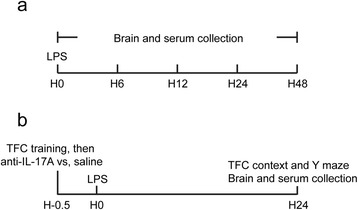
Study design. **a** The hippocampus and serum were collected at 6, 12, 24, and 48 h after LPS injection. **b** Rats were injected with LPS within 30 min after TFC training, and the IL-17A Abs were administrated immediately after TFC training. The hippocampus and serum were collected 24 h after LPS injection. Behavioral tests were also performed at this time point

First, 30 rats were randomly divided into five groups (*n* = 6) as follows: (1) saline (2.5 ml/kg) control group; (2) LPS-treated groups: rats were IP injected with LPS, respectively for 6, 12, 24, and 48 h. Then, 40 rats were randomly divided into four groups (*n* = 10). Two groups were challenged with an acute dose of LPS (*n* = 20; IP administration) while two groups received saline (*n* = 20; IP administration). Of the LPS groups, one group received IL-17A Abs via ICV administration (*n* = 10; anti-IL-17A + LPS group) and a control group received saline also via ICV administration (*n* = 10; LPS group). Similarly with the saline-treated rats, one group received IL-17A Abs through ICV injection (*n* = 10; anti-IL-17A group) while the other group received saline also through an ICV injection (*n* = 10; control group). The study design is briefly illustrated in Fig. 1.

### Cell cultures

Primary rat microglial cells were prepared as previously described, with minor modifications [16]. Briefly, whole brains were isolated from SD rats at postnatal day 1–2. The meninges and blood vessels were removed completely in cold D-Hank’s buffered saline. Next, the brains were minced with sterile scissors and digested with 0.25 % Trypsin-EDTA solution for 10 min at 37 °C. Trypsinization was stopped by adding an equal volume of culture medium, which was high-glucose DMEM containing 10 % FBS and penicillin (100 U/ml)/streptomycin (100 μg/ml). The dissociated cells were passed through a 100-μm-pore mesh, pelleted at 1500 rpm for 5 min, and resuspended in culture medium. The cells were seeded on poly-D-lysine-precoated cell culture flasks and cultured at 37 °C in a humidified atmosphere of 5 % CO_2_/95 % air. After seeding, the medium was replaced every 3–4 days. After the glial cells formed a confluent monolayer (10–14 days), the microglial cells were separated from the astrocytes by shaking for 5 h at 150 rpm. The microglial cells were seeded into 6-well culture plates at a density of 10^5^ cells/cm^2^. After 24 h of culture, the cells were starved overnight and then subjected to treatments. The purity of the microglia was confirmed to be >98 % using immunofluorescence staining for OX-42 (CD11b) and was calculated as follows: number of OX-42 positive cells/number of DAPI positive cells.

### Behavioral analysis

#### Trace fear conditioning (TFC)

TFC was used to assess hippocampal-dependent memory in rodents as previously described [17, 18]. Rats were trained to associate an environment (context) with a conditional stimulus (tone) and an unconditional stimulus (foot shock). The training paradigm was performed as previously described [18]: tone duration, 20 s; level, 80 dB; shock duration, 2 s; and intensity, 0.8 mA. The IP LPS injection was performed 30 min after the fear conditioning paradigm, and IL-17A Abs were given immediately after the fear conditioning paradigm. During training, an initial exploratory phase (100 s) was followed by two trials separated by a 100-s intertrial interval. Trials consisted of a 20-s auditory cue (80 dB, 5 kHz, conditional stimulus), followed by a 2-s foot shock (0.8 mA, unconditional stimulus). Rats anticipate the shock by “freezing,” which is defined as the absence of all movement expect for respiration; this defensive posture reflects learned fear. When placed in the same context on a subsequent occasion, the learned fear is recalled and the amount of learning and recall is measured by the amount of freezing. Contextual memory of the learned fear was assessed 1 day after the LPS injection by returning the rat to the same chamber in which it was trained, in the absence of the tone and shock. Freezing behavior was automatically scored for 300 s by video tracking software (Xeye Fcs, Beijing MacroAmbition S&T Development Co., Ltd., Beijing, China).

#### Y maze

The Y maze consisted of three arms (regions I–III, 30-cm l × 5-cm w × 20-cm h), with the arms at a 120° angle from each other [19]. Each arm had a lamp at the distal end. A safe region was associated with the illumination, whereas the other regions featured electrical foot stimulation (40 ± 5 V). Each rat was first placed at the end of one arm (starting area chosen randomly) and allowed to move freely in the maze during a 3-min session without any stimulation to adapt to the environment. The test was then started, and the illuminated arm (safe region) served as the new starting area. Furthermore, we changed the orientation of the safe and stimulation regions using a randomization method. The test was considered to be successful (learned) if the rat reached the safe region within 10 s. After each foot stimulation, we waited for the rat to reach the illuminated arm (the new starting area) before the next stimulation. If nine responses were correct in 10 consecutive foot stimulations (9/10 standard), the mice were defined as having reached the learning criterion. The total number of stimulations to reach the criterion during training was recorded as the learning ability. All rats reached the learning criterion in the present study.

#### Immunohistochemistry

The cerebral tissues were harvested, fixed with 4 % paraformaldehyde, and then immersed in 15 % sucrose at 4 °C for 24 h followed by 30 % sucrose for 48 h. Sections (10-μm-thick) were prepared. We blocked endogenous peroxidase activity with 3 % H_2_O_2_ in PBS solution for 10 min. Sections were then incubated with the Iba1 polyclonal antibody (1:200) at 4 °C overnight and then incubated with secondary antibody for 2 h. Microglial cells were visualized by adding DAB to the sections. Activated microglia were identified as Iba1-positive cells. For quantification [20, 21], the studied tissue sections were selected with a 150-μm interval according to anatomical landmarks corresponding to Bregma from Bregma −2.8 to −3.8 mm of the rat brain (Paxinos and Watson, 1996). For each animal, 15 photographs from the CA1 area of three hippocampus sections and 15 photographs from the CA3 area of three hippocampus sections were captured using Leika 2500 (Leica Microsystems, Wetzlar, Germany) at 200× magnification. The number of Iba1-positive cells per photograph (0.74-mm^2^ frame) was obtained by using NIH ImageJ software (Bethesda, MD, USA), averaged and converted to cells/mm^2^. Iba1-positive cell counting was performed in a blinded fashion by an experimenter that was unaware of the sample identity.

#### ELISA

The levels of TNF-α and IL-6 in serum, brain tissue extracts, and culture medium were measured with ELISA kits from R&D Systems (Minneapolis, MN, USA). The levels of IL-17A in serum and brain tissue extracts were measured with an ELISA kit from Biolegend (San Diego, CA, USA).

#### Western blotting

Hippocampi were homogenized in RIPA buffer. The homogenates were centrifuged for 15 min at 12,000 g at 4 °C. The quantity of protein in each supernatant was determined using a BCA protein assay kit. Proteins (60 μg) were denatured with sodium dodecyl sulfate (SDS) sample buffer and separated using 10 % SDS-polyacrylamide gel electrophoresis (PAGE). The proteins were transferred to a polyvinylidene fluoride (PVDF) microporous membrane (Millipore, Bedford, MA, USA), which was then blocked with 5 % skim milk for 1 h at room temperature. The membrane was incubated with primary antibody overnight at 4 °C. The following primary antibodies were used: rabbit polyclonal anti-APP, -iNOS, -COX-2, and -BACE1 and mouse monoclonal anti-GAPDH (1:1000). After adding the anti-rabbit or anti-mouse secondary antibody (1:1000) for 1 h, the protein bands on the membranes were detected with ECL kits (Thermo Fisher Scientific, Rockford, IL, USA). The relative density of the protein bands was scanned by densitometry using Image Lab software (Bio-Rad, Richmond, CA, USA) and quantified by NIH ImageJ software (Bethesda, MD, USA).

#### Immunofluorescence

To determine microglial activation, cells were fixed with 4 % paraformaldehyde for 30 min; non-specific binding was blocked by incubating cells in a 5 % BSA and 0.1 % Triton X-100 solution for 1 h at room temperature. The microglial cells were incubated with rabbit anti-Iba1 polyclonal antibody (1:500) in the blocking solution overnight at 4 °C. After three washes with PBS, the microglial cells were incubated with the corresponding FITC-conjugated goat anti-rabbit IgG (1:200) for 2 h at room temperature and the nuclei were stained with DAPI. Fluorescence images were acquired using a Leica TCS SP2 (Leica Microsystems, Buffalo Grove, IL, USA) laser scanning spectral confocal microscope. Quantification was made using the associated Leica LCS software by placing a rectangular region of interest (ROI) across the full image and within the ROI, for every image, mean fluorescence intensity (MFI) was measured and the values were plotted.

#### Statistical analysis

Statistical analyses were performed using GraphPad Prism 5 software (version 5.01, GraphPad Software, San Diego, CA, USA). The results are expressed as the mean ± s.e.m. Data were analyzed with one-way ANOVA followed by Newman-Keuls post hoc test wherever appropriate. A *P* < 0.05 was considered to be a statistical significance.

## Results

### Increases of IL-17A expression induced by LPS

**Fig. 2:**
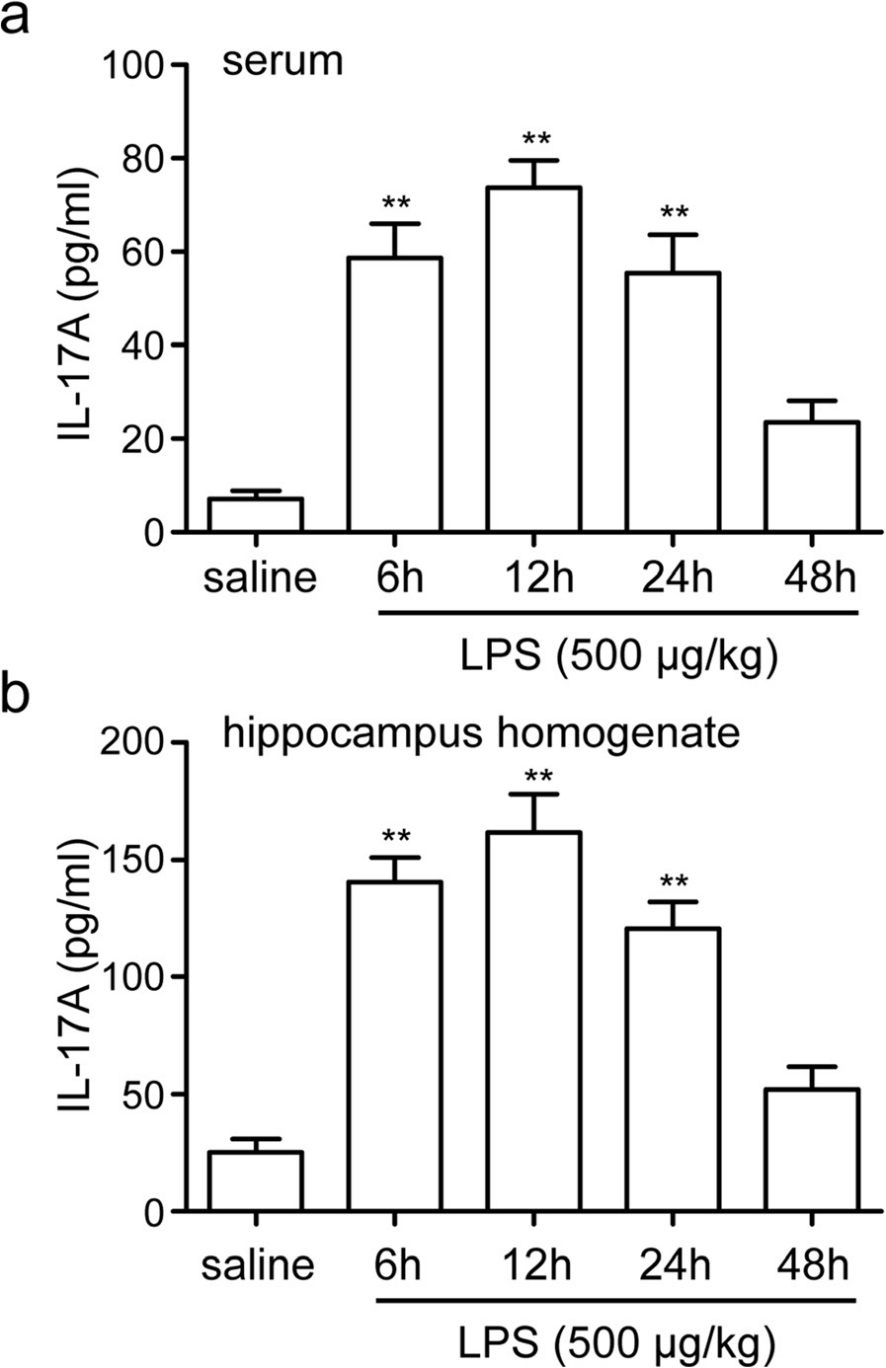
LPS-induced IL-17A expression in serum and in the hippocampus. The rats were equally divided into five groups, the control group and four LPS injection groups, according to four time points: 6, 12, 24, and 48 h after LPS injection. IL-17A protein levels in serum (**a**) and in the hippocampus (**b**) were examined using ELISA. The data are presented as the mean ± s.e.m. (*n* = 6 in **a** and **b**). **P < 0.01 versus saline group

To examine whether IL-17A is involved in LPS-induced neuroinflammation, we studied IL-17A protein expression levels in the serum and in the hippocampus within 48 h after LPS injection. The ELISA data showed that levels of IL-17A in serum and in the hippocampus were significantly higher in the rats challenged by LPS (Fig. 2a, b). The levels of IL-17A in serum significantly increased at 6 h after LPS administration, reached the peak point at 12 h, and remained elevated at 24 h, as compared with rats receiving saline (F4, 25 = 20.97, *P* < 0.001; saline group 7.04 ± 1.73 pg/ml, 6-h group 58.61 ± 7.37 pg/ml, 12-h group 73.80 ± 5.70 pg/ml, 24-h group 55.35 ± 8.29 pg/ml, *P* < 0.01, Fig. 2a). Similar effect was also observed in the hippocampus for LPS-increased IL-17A expression that occurred at 6, 12, and 24 h of stimulation, respectively, when compared with the saline group (F4, 25 = 26.89, *P* < 0.001; saline group 25.13 ± 5.62 pg/ml, 6-h group 140.41 ± 10.59 pg/ml, 12-h group 161.66 ± 16.33 pg/ml, 24-h group 120.50 ± 11.55 pg/ml, *P* < 0.01, Fig. 2b). These results indicate that IL-17A may be involved in LPS-induced neuroinflammation.

### IL-17A Abs improve LPS-induced memory impairment

**Fig. 3:**
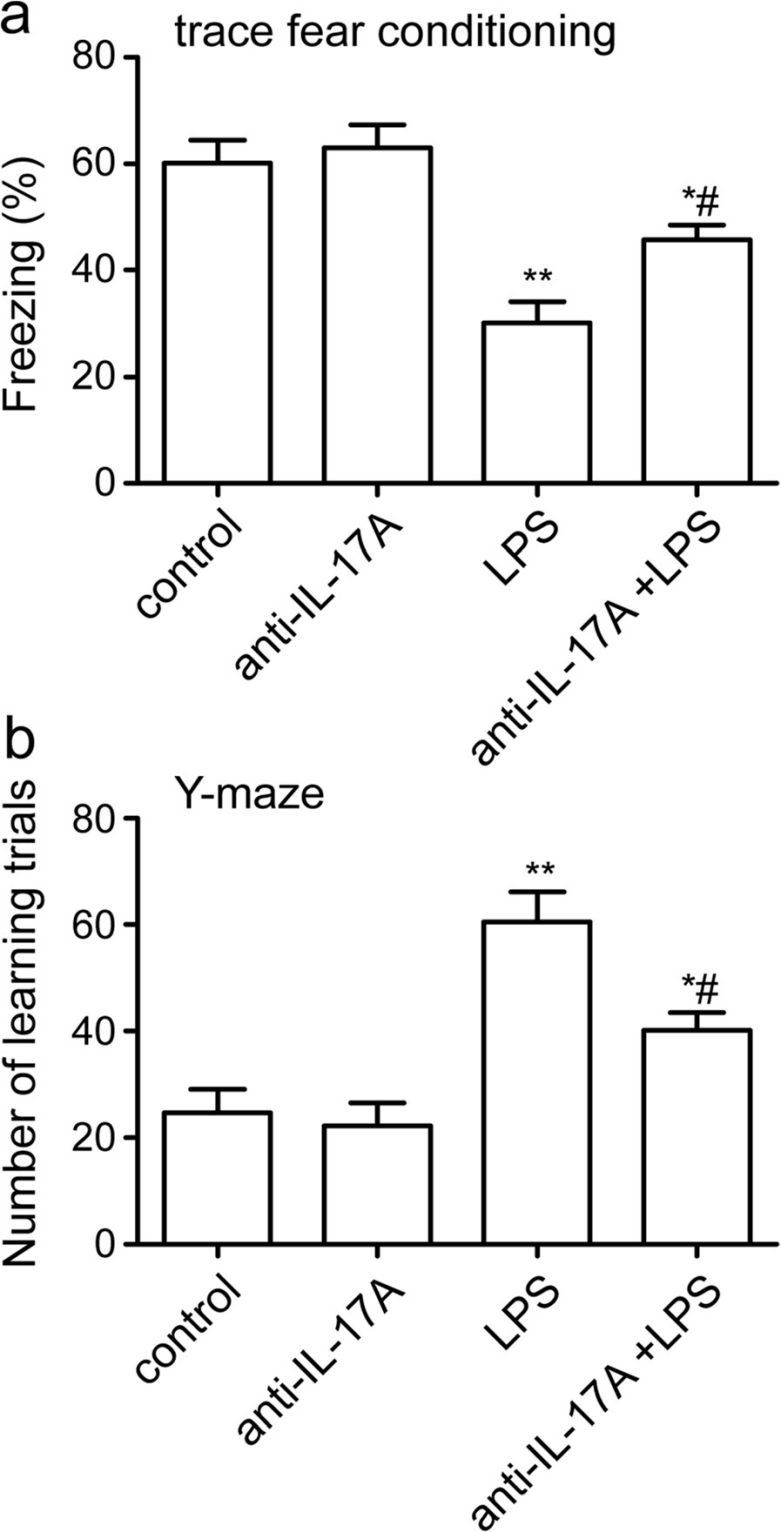
IL-17A Abs improved LPS-induced memory impairment. **a** Contextual fear response, as measured by freezing behavior, was determined in the rats. **b** The Y-maze test was performed after TFC in the rats. The data are presented as the mean ± s.e.m. (*n* = 6 in **a** and **b**). *P < 0.05, **P < 0.01 versus control group. ^#^ *P* < 0.05 versus LPS treatment group

To further evaluate the role of IL-17A on the LPS-induced memory impairment model, rats were injected with IL-17A Abs 30 min prior to LPS injection. One day after the LPS injection, we performed contextual assessment and the Y-maze test to observe the cognitive function of the rats. As shown in Fig. 3a, b, the rats exposed to LPS exhibited a significant reduction in cognitive function compared to animals exposed only to saline (freezing: F3, 20 = 14.92, *P* < 0.001; LPS group 30.00 ± 4.04 versus control group 60.17 ± 4.28, number of learning trials: F3, 20 = 15.19, *P* < 0.001; LPS group 60.50 ± 5.69 versus control group 24.67 ± 4.36, *P* < 0.01). In an attempt to ameliorate this LPS-induced cognitive impairment, we injected IL-17A Abs 30 min before LPS injection. Treatment with IL-17A Abs significantly improved freezing behavior and the number of learning trials, indicating it attenuated the memory dysfunction caused by LPS (freezing anti-IL-17A + LPS group 45.67 ± 2.80, number of learning trials anti-IL-17A + LPS group 40.17 ± 3.36, *P* < 0.05, Fig. 3a, b). Together, these results indicate that IL-17A is involved in LPS-induced memory impairment and suggest a use for IL-17A Abs in limiting the adverse cognitive outcomes caused by endotoxemia.

### IL-17A Abs inhibit the hippocampal TNF-α and IL-6 expression induced by LPS

**Fig. 4:**
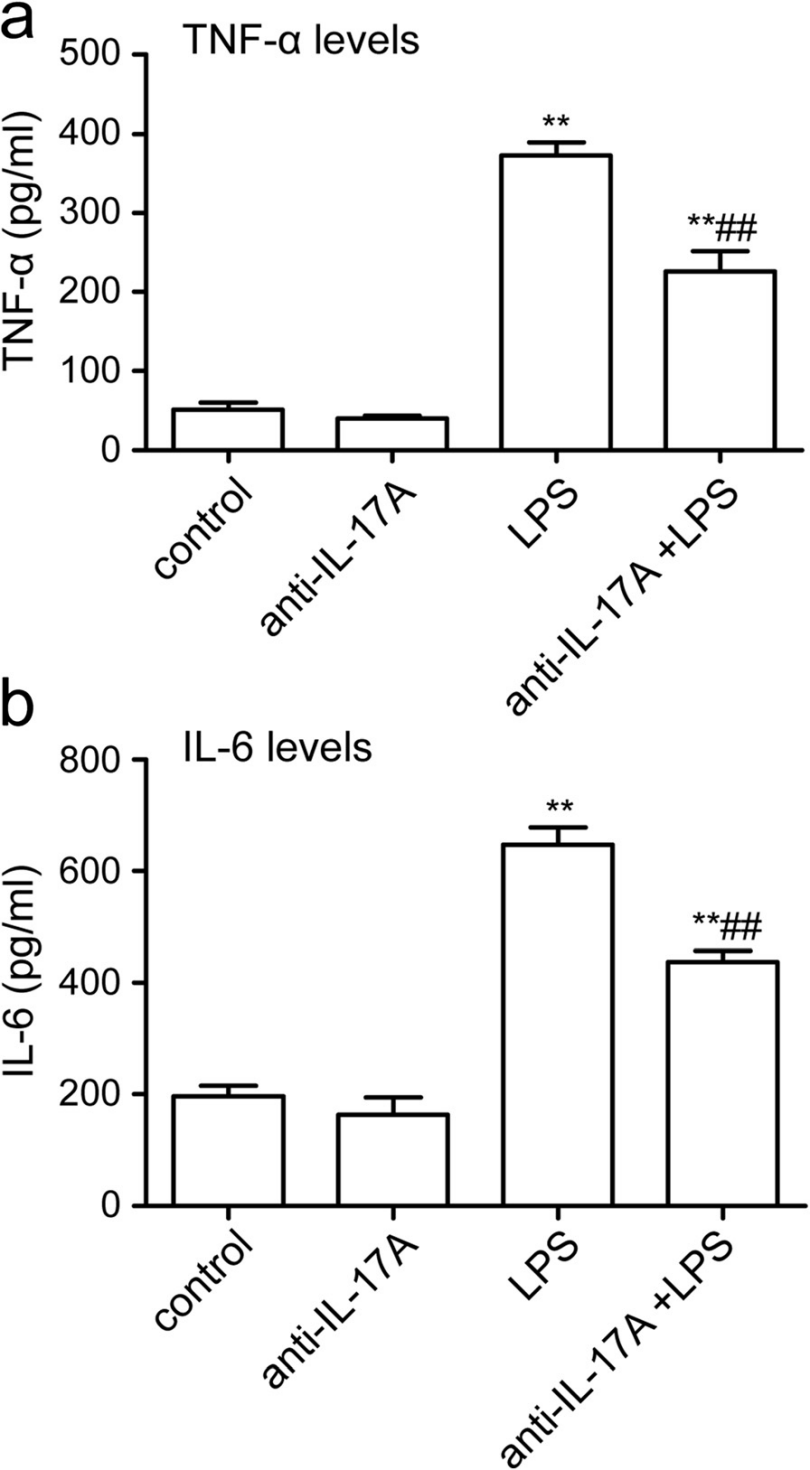
IL-17A Abs inhibited the hippocampal TNF-α and IL-6 expression induced by LPS. TNF-α and IL-6 protein expression in serum (**a**) and in the hippocampus (**b**) were determined by ELISA. The data are presented as the mean ± s.e.m. (*n* = 6 in **a** and **b**). **P < 0.01 versus control group, ^##^ *P* < 0.01 versus LPS treatment group

To determine whether the IL-17A Abs could suppress LPS-induced neuroinflammation in aged rats, protein levels of TNF-α and IL-6 in the hippocampus were examined by ELISA. As shown in Fig. 4a, b, following LPS injection for 1 day, levels of TNF-α and IL-6 in the hippocampus significantly increased by up to approximately 731 and 329 % of the control values, respectively (TNF-α LPS group 372.69 ± 16.64 pg/ml versus control group 50.99 ± 9.19 pg/ml, IL-6 LPS group 647.91 ± 30.38 pg/ml versus control group 196.66 ± 19.21 pg/ml, *P* < 0.01). Pre-treatment with IL-17A Abs for 30 min partially abolished the increase in LPS-induced TNF-α and IL-6 production (TNF-α: F3, 20 = 97.89, *P* < 0.001; anti-IL-17A + LPS group 226.04 ± 25.49 pg/ml, IL-6: F3, 20 = 78.14, *P* < 0.001; anti-IL-17A + LPS group 437.99 ± 18.82 pg/ml, *P* < 0.01, Fig. 4a, b). These results suggest that IL-17A Abs can downregulate LPS-induced neuroinflammation.

### IL-17A Abs reduce the LPS-induced increase in Iba1-positive cells in hippocampal area CA1 and CA3

**Fig. 5:**
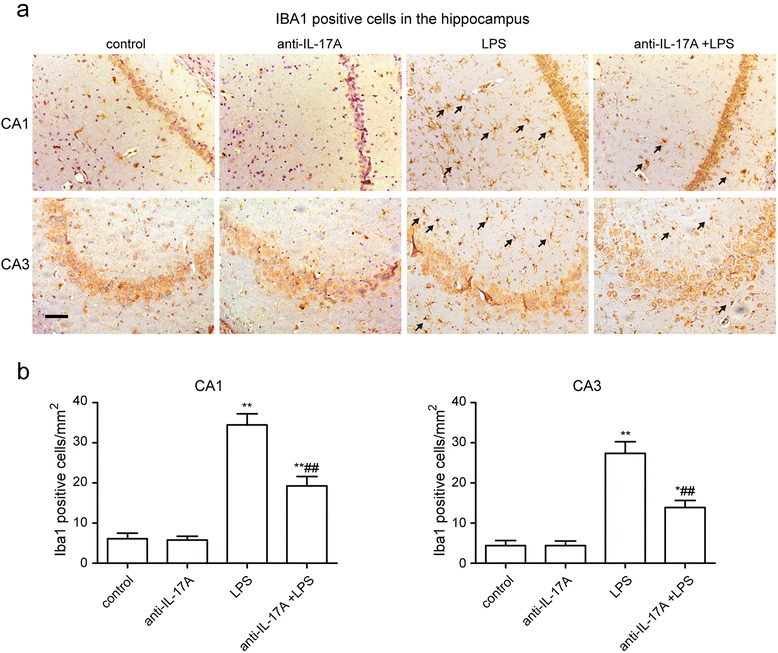
IL-17A Abs reduced the LPS-induced increase in Iba1-positive cells in hippocampal area CA1 and CA3. Hippocampal sections (10-μm) were prepared 24 h after the LPS injection. **a** Representative immunohistochemistry graphs of microglia in area CA1 and CA3 of the hippocampus. **b** Quantification of Iba1-positive cells in area CA1 and CA3 of the hippocampus. Graphs show the mean ± s.e.m. (*n* = 4). *P < 0.05, **P < 0.01 versus control group, ^##^ *P* < 0.01 versus LPS treatment group. Bar = 50 μm

Coincident with the change in TNF-α and IL-6 expression in the hippocampus, LPS injection also induced an increase in Iba1-positive cells in area CA1 and CA3 of the hippocampus (CA1: F3, 12 = 44.36, *P* < 0.001; LPS group 25.5 ± 2.1 versus control group 4.5 ± 1.04, *P* < 0.01, CA3: F3, 12 = 28.39, *P* < 0.001; LPS group 20.25 ± 2.18 versus control group 3.25 ± 0.95, *P* < 0.01, Fig. 5a, b). The microglia exhibited enlarged cytoplasm and cell bodies, irregular shapes, and intensified Iba1 staining, consistent with the morphological characteristics of activated microglia (Fig. 5a). This effect was significantly inhibited by the IL-17A Abs (CA1: anti-IL-17A + LPS group 14.25 ± 1.75, *P* < 0.01, CA3: anti-IL-17A + LPS group 10.25 ± 1.32, *P* < 0.01, Fig. 5a, b), suggesting that IL-17A Abs could suppress the microglial activation induced by LPS injection.

### IL-17A Abs inhibit COX-2, iNOS, BACE1, and APP expression and increase the expression of PSD95 in the aged rats treated with LPS

**Fig. 6:**
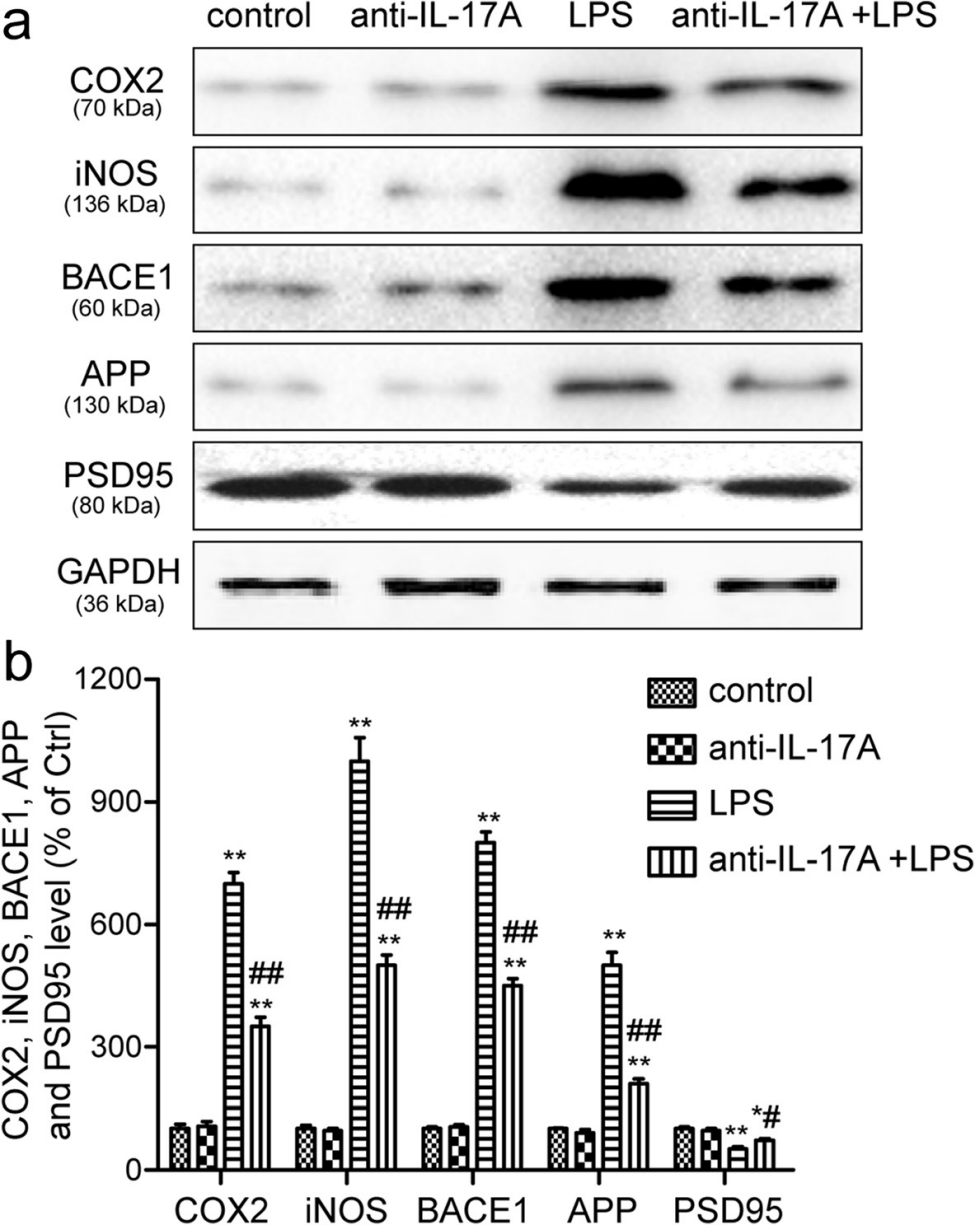
IL-17A Abs inhibit COX-2, iNOS, BACE1, and APP expression and increase the expression of PSD95 in the aged rats treated with LPS. **a** The expression of COX-2, iNOS, BACE1, APP, and PSD95 was detected by Western blotting using specific antibodies in the hippocampus of rats. Each blot is representative of three experiments. **b** Levels of COX-2, iNOS, BACE1, APP, and PSD95 were quantified and normalized to GAPDH levels. Each value was then expressed relative to the control, which was set to 1. The data are presented as the mean ± s.e.m. **P < 0.01 versus control group, ^##^ *P* < 0.01 versus LPS treatment group

To investigate the inhibitory effect of the IL-17A Abs on memory impairment via inhibition of neuroinflammation, COX-2 and iNOS expression in the hippocampus were also determined by Western blot analysis. Upon LPS treatment, the expression of COX-2 and iNOS in the hippocampus of LPS-injected rats was significantly higher than the expression in control rats, but these elevations were remarkably inhibited by the IL-17A Abs (Fig. 6a, b). BACE1 and APP play a key role in the process of amyloidogenesis. Therefore, we examined the expression of BACE1 and APP in the hippocampus. Western blot analysis showed that BACE1 and APP expression were significantly increased by LPS injection in the rat brains, whereas densitometry data showed that LPS-induced BACE1 and APP expression were remarkably inhibited by the IL-17A Abs (Fig. 6a, b). The expression of synaptic marker PSD95 was also evaluated in the present study. We found that IL-17A Abs could increase the expression of PSD95 in the aged rats treated with LPS (Fig. 6a, b).

### Effects of IL-17A on microglial activation and cytokine production

**Fig. 7:**
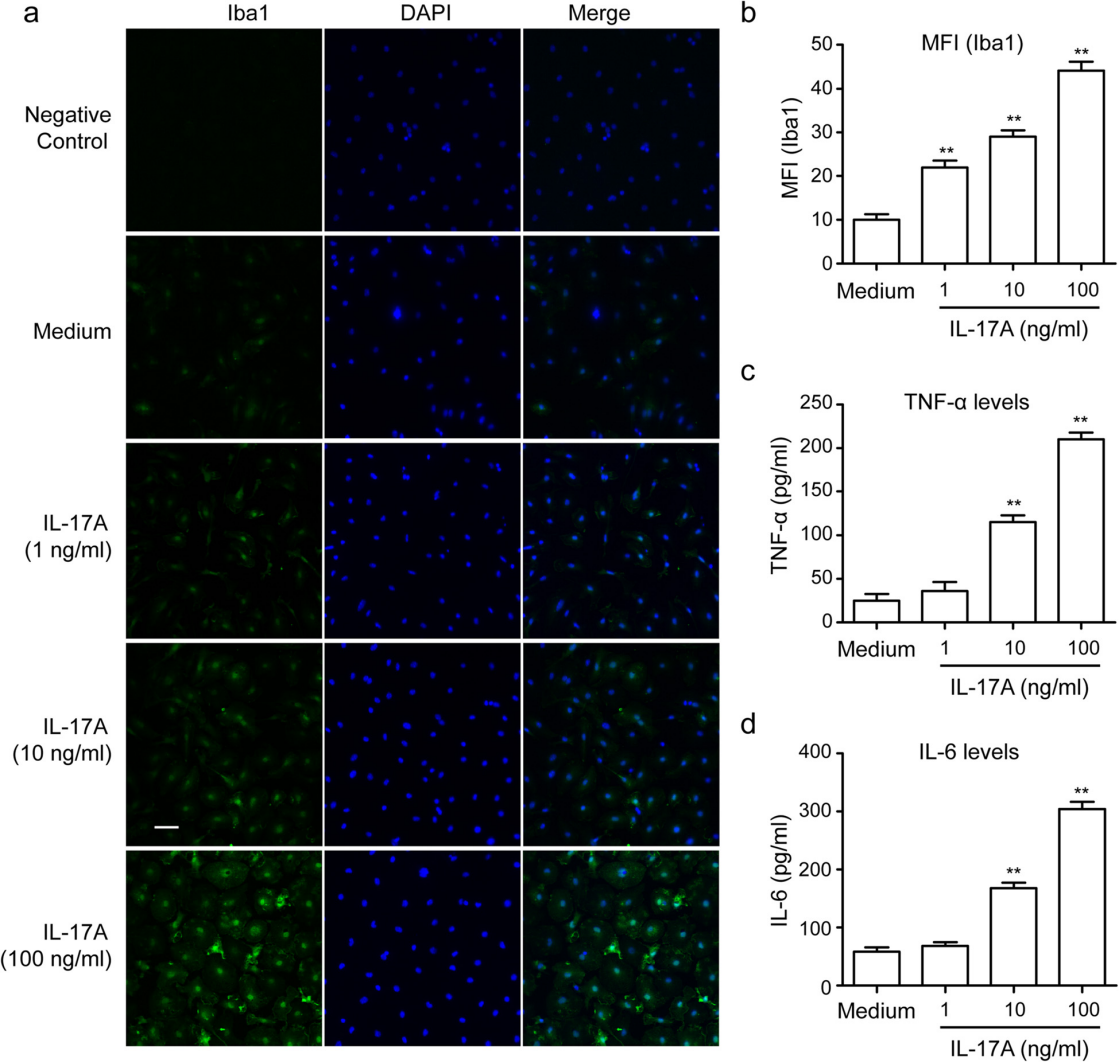
Effects of IL-17A on microglial activation and cytokine production. Primary microglial cells were incubated with IL-17A at 1, 10, and 100 ng/ml for 24 h. **a** The cells were stained with an Iba1 antibody. Upregulated Iba1 expression (*green*) in activated microglia was observed using confocal scanning. The *blue staining* represents DAPI. Scale bar = 50 μm. **b** Graph showing the mean fluorescence intensity (MFI) for Iba1. **c**, **d** Quantification of TNF-α and IL-6 in the media. The data are presented as the mean ± s.e.m. of four independent experiments. **P < 0.01 versus the response to medium alone

Microglia play a pivotal role in neuroinflammation. To investigate whether IL-17A could stimulate microglial activation, microglia were cultured with various concentrations of IL-17A (1, 10, and 100 ng/ml) for 24 h. Immunofluorescence analysis showed that IL-17A at a concentration of 10 ng/ml or greater remarkably increased Iba1 expression in microglia, suggesting that IL-17A can induce microglial activation (MFI: F3, 12 = 79.06, *P* < 0.001; medium group 10.01 ± 1.24, 1 ng/ml group 21.94 ± 1.61, 10 ng/ml group 29.07 ± 1.40, 100 ng/ml group 44.10 ± 2.04, Fig. 7a, b). Microglia-mediated neuroinflammation occurs primarily due to excessive pro-inflammatory mediators and their downstream signaling cascades. Levels of pro-inflammatory mediators were determined in the present study. As shown in Fig. 7c, d, after incubation with various concentrations of IL-17A for 24 h, the production of TNF-α and IL-6 from primary microglial cells significantly increased at an IL-17A concentration of 10 ng/ml or greater (TNF-α: F3, 12 = 102.3, *P* < 0.001; medium group 24.89 ± 7.79 pg/ml, 10 ng/ml group 115.32 ± 7.55 pg/ml, 100 ng/ml group 210.30 ± 7.78 pg/ml, IL-6: F3, 12 = 149.8, *P* < 0.001; medium group 58.39 ± 7.94 pg/ml, 10 ng/ml group 168.44 ± 9.04 pg/ml, 100 ng/ml group 304.25 ± 12.85 pg/ml, *P* < 0.01), suggesting that IL-17A can upregulate the production of inflammatory factors.

## Discussion

The role of IL-17A in neurodegenerative diseases such as MS has been widely confirmed [22–24]; however, little is known about whether IL-17A is involved in LPS-induced neuroinflammation and cognitive impairment. In this paper, we demonstrated that LPS could induce IL-17A expression in the CNS and that IL-17A Abs, which neutralize IL-17A, suppressed neuroinflammation via the inhibition of microglial activation in an LPS-induced in vivo model and ameliorated memory impairment. In vitro, we found that IL-17A could stimulate microglial activation and the production of pro-inflammatory cytokines.

It is well known that LPS can induce the production of inflammatory cytokines, and LPS-induced systemic inflammation in rats is frequently used as a model for studying neuroinflammation and cognitive impairment. The release of pro-inflammatory cytokines, such as TNF-α and IL-6, has been implicated in LPS-induced systemic inflammation. To data, intensive studies have been carried out regarding the potential pro-inflammatory properties of IL-17A; for example, IL-17A seems to be important in sepsis [25]. Flierl et al. found that the levels of IL-17A in mice rose time dependently in plasma after cecal ligation and puncture (CLP), however, neutralization of IL-17A by the antibodies improved sepsis (survival from ~10 to nearly 60 %), which were associated with substantially significant reductions of systemic pro-inflammatory cytokines and chemokines in plasma. In the present study, we found that LPS could increase the expression of IL-17A in serum. Interestingly, the levels of IL-17A were also found increasing in the hippocampus.

Recent studies have shown that IL-17A may play a role in cognitive dysfunction [26–28]. IL-17A was associated with poorer cognitive status in subjects with depressive symptoms in ischemic stroke patients [26]. McManus et al. found that respiratory infection could promote the infiltration of IL-17-producing T cells in older APP/PS1 mice, which was accompanied by increased glial activation and amyloid-β deposition [28]. Amyloid-β injection also could increase the expression of IL-17A in the hippocampus, accompanied with spatial memory impairment in rats [29]. Our results showed that the freezing behavior of animals treated with LPS was decreased, whereas pretreatment with IL-17A Abs was able to partially reverse the cognitive deficits seen following LPS treatment. The freezing behavior indicates that the rats recalled the learned fear when placed in the same context [30]. Consistent with the TFC results, the number of learning trials was increased in animals treated with LPS in Y maze; however, it was decreased in animals pre-treated with IL-17A Abs compared to the LPS-alone group. Therefore, the data presented here show that IL-17A Abs administration could prevent the cognitive deficits seen in LPS-treated animals. A previous report may support our observation above. In a surgery model, Tian et al. found that partial hepatectomy also could increase the levels of IL-17A in the hippocampus and induce cognitive impairment in mice, while vitamin D ameliorated cognitive dysfunction through inhibiting Th17 cells accompanied with expansion in Treg cells [27]. These in vivo experiments indicate that IL-17A may be involved in LPS-induced cognitive impairment and may play a detrimental role in it.

Cognitive decline is prominent in Alzheimer’s disease (AD) and also occurs in other disorders in which neuroinflammation is believed to play a prominent role [31]. Thus, the next step was to evaluate the effects of the IL-17A Abs on the neuroinflammatory processes induced by LPS administration. Recently, several researchers were reported that systemic administration of LPS induces release of pro-inflammatory mediators and cytokines such as TNF-α, IL-6, iNOS, and COX-2 in the brain, indicating that systemic inflammation induces neuroinflammation [32]. Moreover, systemic administration of LPS has been reported to result in increased APP processing as well as memory deficiency with concomitant increased neuroinflammation [33]. Administration of non-steroidal anti-inflammatory drugs (NSAIDs) could reduce the risk and delay the onset of AD [34, 35]. Thus, anti-inflammation could decrease memory deficiency via the prevention of neuroinflammation. In the present study, we found that pre-treatment with IL-17A Abs significantly inhibited the LPS-induced expression of TNF-α, IL-6, iNOS, and COX-2 in the hippocampus. APP is the source of extracellular amyloid-β plaques, which are believed to cause damage to neurons, especially to neuronal synapses [36]. BACE1 is the rate-limiting enzyme for the formation of amyloid-β [37]. Expression of APP and BACE1 has been shown to be increased in neuroinflammation and to be involved in cognitive impairment [38]. We found that pre-treatment with IL-17A Abs significantly inhibited the LPS-induced expression of APP and BACE1, increased LPS-induced decline of PSD-95, alleviated neuronal synapses damage. IL-17A is believed to have a particular role in the delayed phase of the post-infarct inflammatory cascade. A significantly higher number of IL-17A-expressing cells in ischemic tissue has been detected in postmortem studies in both humans and rodents [39, 40]. Shichita et al. demonstrated that IL-17A-deficient mice showed a reduction in infarct volumes and levels of TNF-α and IL-1β in the brain [41]. Zong et al. found that IL-17A could promote spinal cord neuroinflammation after spinal cord injury [42]. Overexpression of IL-17A in astrocytes could lead to increase in LPS-induced neuroinflammation in vivo [43]. While blocking Th-17 cells trafficking attenuates neuroinflammation after LPS/hypoxic-ischemic [44]. Taken together, these in vivo experiments point to a detrimental role of IL-17A in LPS-induced neuroinflammation and inhibition of IL-17A expression could partially prevent neuroinflammation.

Microglia, important immune cells in the CNS, are regarded as the tissue macrophages of the brain. Within the aged brain, microglia are primed and easily produce a more violent response to inflammatory stimulation [45]. Microgliosis, which is defined as an increased number of microglia, is an important response of neuroinflammation [46]. In our in vivo study, the number of Iba1-positive cells in hippocampal area CA1 and CA3 increased in aged rats subjected to LPS, which is consistent with the overproduction of pro-inflammatory cytokines and proteins in the hippocampus and with the sharp decline in behavioral performance. IL-17A Abs pre-treatment reversed the hippocampal microgliosis induced by LPS. These results indicate that IL-17A may be involved in LPS-induced microglial activation. To investigate whether IL-17A can induce microglial activation or not, we used recombinant IL-17A protein to stimulate microglia for 24 h in vitro. We found that IL-17A could induce microglial activation and increase the expression of pro-inflammatory cytokines in microglia in a dose-dependent manner. A previous report that LPS significantly induced IL-17A expression in BV-2 microglial cell line may support our observation above [47]. The study by Kawanokuchi et al. demonstrated that microglia express the IL-17A receptor. They also showed that IL-17A could upregulate the expression of IL-6 mRNA in microglia in vitro [48]. Murphy et al. also found that myelin oligodendrocyte glycoprotein (MOG)-induced IL-17A-producing Th1/Th17 cells could stimulate microglial activation and pro-inflammatory cytokine production; however, MOG-induced IFN-γ-producing Th1 cells could not stimulate the activation of microglia in vitro [49]. Zimmermann et al. have also shown that overexpression of IL-17A in astrocytes led to microglial activation either following LPS challenge or not in vivo [43]. These findings indicate that IL-17A could induce microglial activation, which have been confirmed to play a key role in neurodegenerative diseases and cognitive impairment [45]. However, another study by Prajeeth et al. showed that effector molecules released by Th1 but not Th17 cells drive an M1 response in microglia in vitro [50]. This study could not demonstrate that IL-17A has no effect on microglial activation, because both Th1 and Th17 cells could produce various cytokines, which achieved a complex effect on microglia.

## Conclusions

In conclusion, our results suggest that IL-17A is involved in LPS-induced neuroinflammation and cognitive impairment in aged rats via microglial activation. Anti-IL-17A may be a new therapeutic strategy for the treatment of endotoxemia-induced neuroinflammation and cognitive dysfunction.

## Additional file

Additional file 1: Figure S1. The specificity of IL-17A antibody to IL-17. The antibodies were incubated with blocking peptide (BL) 10, 20, and 40 μg/μl, respectively, before injection. TNF-α protein expression in the hippocampus was determined by ELISA. The data are presented as the mean ± s.e.m. (*n* = 3). ***P* < 0.01 versus control group, ^##^ *P* < 0.01 versus LPS treatment group, &&*P* < 0.01 versus LPS treatment group, ^^*P* < 0.01 versus LPS + anti-IL-17A group. **Figure S2.** The full gels of western blots. (DOCX 910 kb)
