# Supplementary material for: IL-17A is implicated in lipopolysaccharide-induced neuroinflammation and cognitive impairment in aged rats via microglial activation
Source: J Neuroinflammation. 2015 Sep 15;12:165. doi: 10.1186/s12974-015-0394-5 (PMC4572693; doi:10.1186/s12974-015-0394-5)
Supplement: Additional file 1: Figure S1. — The specificity of IL-17A antibody to IL-17. The antibodies were incubated with blocking peptide (BL) 10, 20, and 40 μg/μl, respectively, before injection. TNF-α protein expression in the hippocampus was determined by ELISA. The data are presented as the mean ± s.e.m. (n = 3). **P < 0.01 versus control group, ## P < 0.01 versus LPS treatment group, &&P < 0.01 versus LPS treatment group, ^^P < 0.01 versus LPS + anti-IL-17A group. Figure S2. The full gels of western blots. (DOCX 910 kb) [file 12974_2015_394_MOESM1_ESM.docx]

Supplement Figure 1


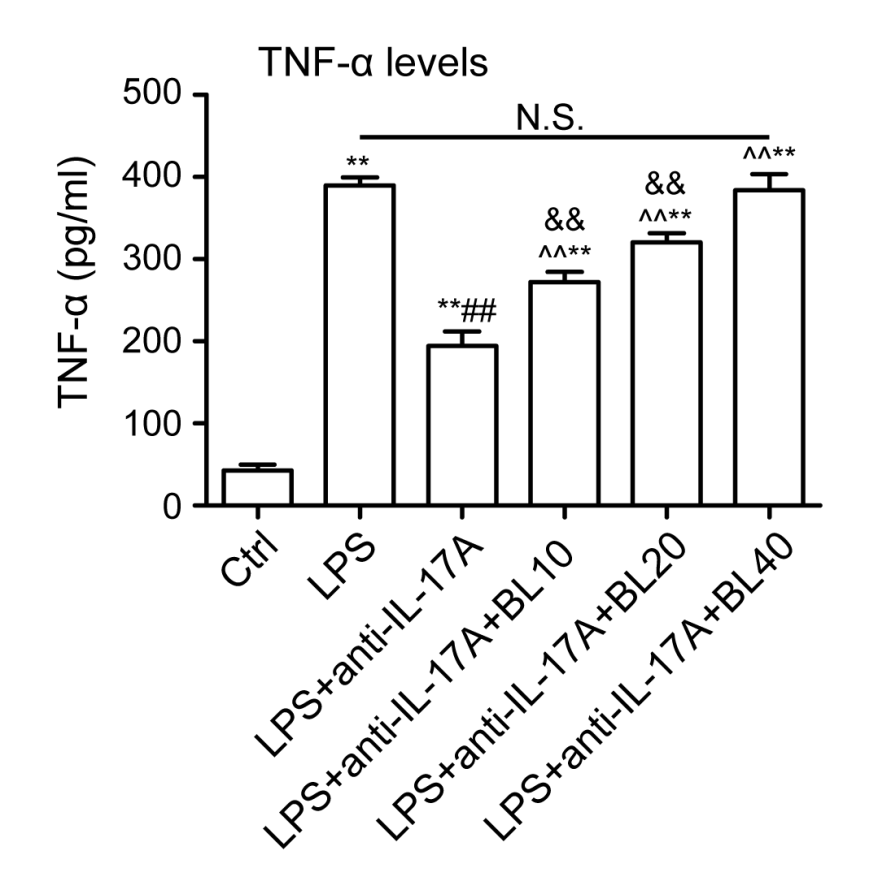


Supplement Figure 1. The specificity of IL-17A antibody to IL-17. The antibodies were incubated with blocking peptide (BL) 10 μg/μl, 20μg/μl and 40μg/μl, respectively, before injection. TNF-α protein expression in the hippocampus were determined by ELISA. The data are presented as the mean ± s.e.m. (n = 3). ** *P* < 0.01 versus Ctrl group, ## *P* < 0.01 versus LPS treatment group, ^&&^ *P* < 0.01 versus LPS treatment group, ^^ *P* < 0.01 versus LPS+anti-IL-17A group.

Supplement Figure 2


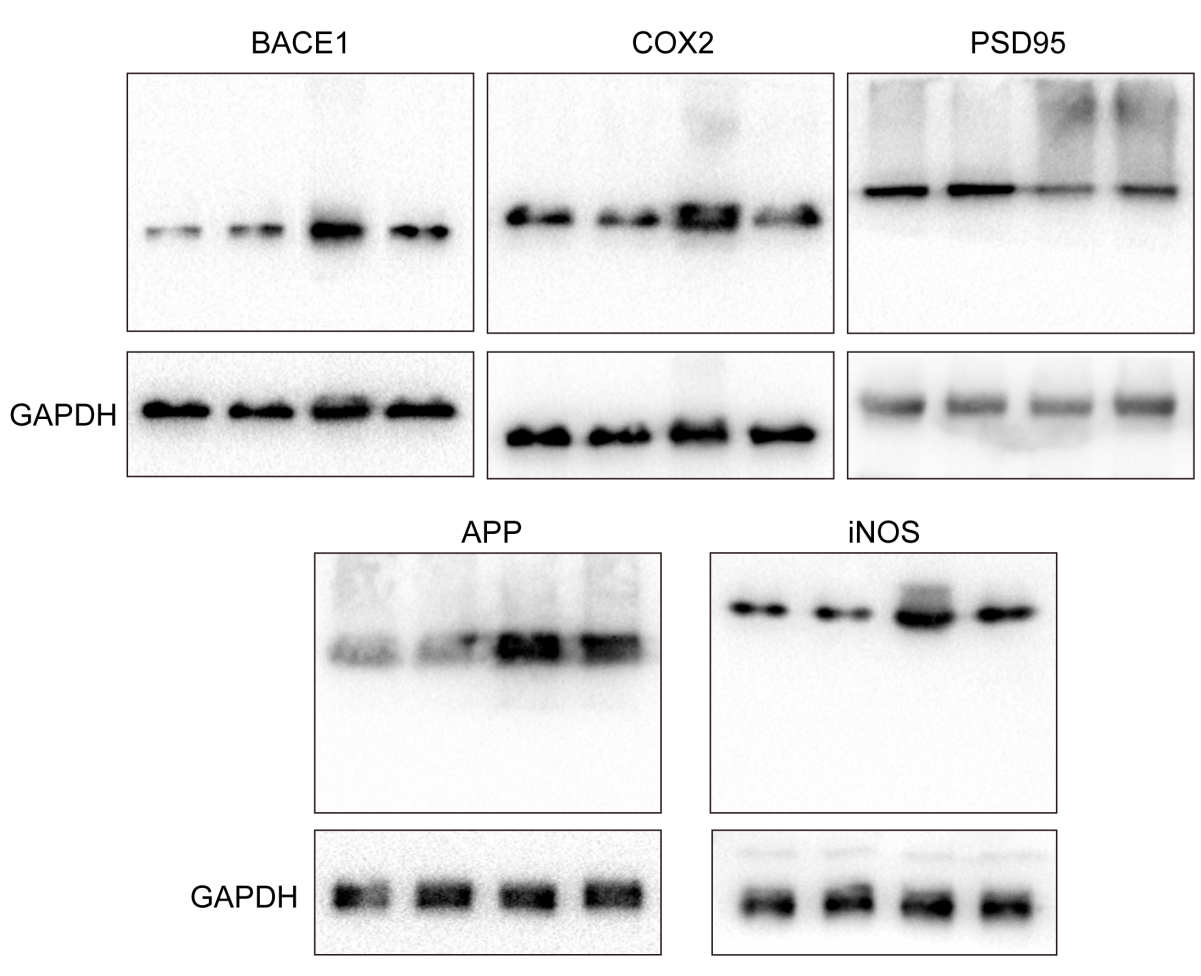


Supplement Figure 2. The full gels of western blors.
